# Prognostic value of transient ischemic dilatation by ^13^N-ammonia PET MPI for short-term outcomes in patients with non-obstructive CAD

**DOI:** 10.1007/s12149-024-01976-8

**Published:** 2024-09-09

**Authors:** Yanni jia, Yingqi Hu, Lihong Yang, Xin Diao, Yuanyuan Li, Yanhui Wang, Ruonan Wang, Jianbo Cao, Sijin Li

**Affiliations:** 1 Department of Nuclear Medicine, First Hospital of Shanxi Medical University, Taiyuan, Shanxi China; 2 Collaborative Innovation Center for Molecular Imaging of Precision Medicine, Shanxi Medical University, Taiyuan, Shanxi China; 3 School of Forensic Medicine, Shanxi Medical University, Taiyuan, Shanxi China; 4 Shanxi Key Laboratory of Molecular Imaging, Shanxi Medical University, Taiyuan, Shanxi China

**Keywords:** Transient ischaemic dilatation, Non-obstructive coronary artery disease, ^13^N-ammonia PET, Myocardial perfusion imaging, Prognosis

## Abstract

**Objective:**

Transient ischaemic dilatation (TID) had incremental diagnostic and prognostic value in obstructive coronary artery disease (CAD), but its clinical significance in patients with non-obstructive CAD remains unknown. We aimed to explore the prognostic value of TID in patients with non-obstructive CAD by ^13^N-ammonia PET imaging.

**Methods:**

We retrospectively studied 131 consecutive patients with non-obstructive CAD undergoing one-day rest-stress ^13^N-ammonia PET myocardial perfusion imaging (MPI). TID was automatically generated using CardIQ Physio software. The receiver operative characteristic (ROC) curve was used to determine the optimal threshold of TID. The follow-up outcome was major adverse cardiac events (MACE), a composite of re-hospitalization for heart failure or unstable angina, late revascularization, non-fatal myocardial infarction, and cardiac death. Cardiac event-free survivals for normal and abnormal TID were compared using Kaplan–Meier plots and log-rank tests.

**Results:**

During a median follow-up of 42.08 ± 17.67 months, 22 (16.7%) patients occurred MACE. The optimal cut-off value of TID was 1.03 based on MACE. Our preliminary outcome analysis suggests that TID-abnormal subjects had a lower overall survival probability. Furthermore, our multivariate analysis reveals abnormal TID was the only independent predictor for MACE in non-obstructive CAD. In the subgroup analysis, an abnormal TID was an independent predictor for MACE in patients with abnormal perfusion patterns.

**Conclusion:**

Among patients with non-obstructive CAD, PET-derived TID ≥ 1.03 may identify those with a high risk of subsequent MACE independently. It was also an independent risk factor for poor prognosis in patients with abnormal perfusion.

**Graphical abstract:**

*CAD* coronary artery disease,* PET* positron emission tomography, *MPI* myocardial perfusion imaging, *TID* transient ischaemic dilatation,* MACE* major adverse cardiac events, *ROC* receiver operative characteristic.
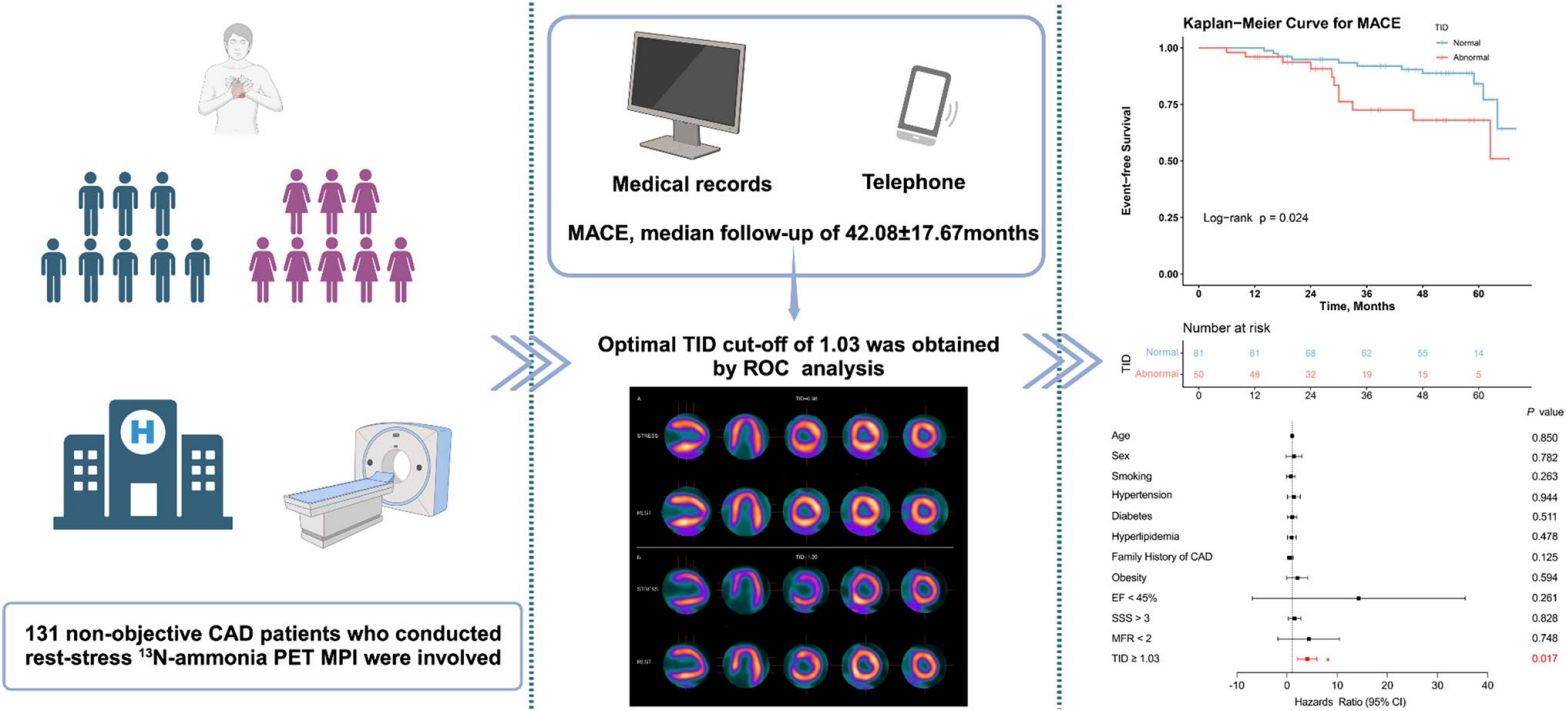

**Supplementary Information:**

The online version contains supplementary material available at 10.1007/s12149-024-01976-8.

## Introduction

Transient ischemic dilation (TID) is recognized as an increased left ventricular (LV) cavity size post-stress, which has been used as a marker for the detection of obstructive coronary artery disease (CAD) [1]. TID derived from single photon emission computed tomography (SPECT) myocardial perfusion imaging (MPI) enhances the diagnostic accuracy of SPECT for patients of CAD [2–4] and has been confirmed as an independent predictor of severe ischemia and poor prognosis in patients with obstructive CAD [5]. However, it is still challenging the detection of coronary microvascular dysfunction (CMD) in non-obstructive CAD which may also lead to an increase in TID. Benefiting from its superior quality of images and significant advantage in quantification analysis, positron emission tomography (PET) has shown a higher diagnostic accuracy in cardiovascular disease [6]. Employing ^13^N-ammonia, PET TID has demonstrated its ability to reflect the degree of coronary microvascular dysfunction in hypertrophic heart disease [7, 8] and thus conveys potential value in non-obstructive CAD, which mainly results from CMD [9]. Nevertheless, the value of PET TID in identifying non-obstructive CAD and prediction of major adverse cardiac events (MACE) is yet to be evaluated.

In this study, We aimed to investigate the prognostic value of TID obtained using ^13^N-ammonia PET in patients with non-obstructive CAD. The correlation between TID and LV function, perfusion defect scores, and absolute coronary flow was also investigated.

## Materials and methods

### Study population and design

We retrospectively included consecutive patients who underwent rest-stress ^13^N-ammonia PET MPI at the Department of Nuclear Medicine, First Hospital of Shanxi Medical University between March 2017 and November 2022, based on their clinical indications. This study was granted approval from the Ethics Review Committee of the First Hospital of Shanxi Medical University and all patients provided informed consent. This study focused on patients with symptoms of myocardial ischemia but without obstructive stenosis of the coronary lumen as confirmed by previous coronary arteriography (CAG) and coronary computed tomography angiography (CCTA). They underwent ^13^N-ammonia PET MPI for further assessment of coronary microvascular function after CAG or CCTA. Thus, the study participants were required to meet specific criteria: (1) non-obstructive CAD, characterized by negative or < 50% luminal stenosis on CAG or CCTA; (2) with reliable PET MPI data. The exclusion criteria were: (1) individuals with obstructive CAD, with ≥ 50% luminal stenosis on CAG or CCTA, and no test results available; (2) individuals with a history of percutaneous coronary intervention (PCI) or coronary artery bypass grafting (CABG); (3) patients with organic heart disease such as myocarditis, cardiomyopathy, and valvulopathy; (4) patients with cancer; (5) patients who did not have the follow-up results. We collected initial information including age, sex, body mass index (BMI), as well as cardiovascular risk factors like smoking, diabetes, hypertension, dyslipidemia, and family history of CAD and medication usage.

### ^13^N-*Ammonia* PET imaging acquisition

PET image acquisition was performed with a GE Discovery VCT PET/CT System (GE Healthcare, Waukesha, Wisconsin), with the CT component comprising a 64-slice CT scanner. Patients were instructed to abstain from all caffeine-containing substances for 24 h, fast for 4 h, and pause beta-blockers, calcium channel blockers, and nitrates on the day of the test.

For ^13^N-ammonia PET MPI, a one-day rest-stress protocol was used, as described previously [10, 11]. Vasodilatory stress was initiated by administering dipyridamole or adenosine at least 40 min after the injection of the rest dose. An intravenous injection of approximately 370 MBq (10 mCi) of ^13^N-ammonia was administered, followed by 10 min and 20 s of PET acquisition in two-dimensional list mode. A low-dose CT scan was obtained before each myocardial perfusion protocol for attenuation correction. Heart rate, blood pressure, and 6 lead electrocardiography (ECG) were recorded during the entire scan.

### Analysis of perfusion and gated images

PET images were reconstructed using ordered-subset expectation maximization reconstruction (OSEM) with 2 iterations and 20 subsets according to standard clinical protocols The list-mode data was ultimately resampled to generate gated (8 bins per cardiac cycle) and dynamic images (16 frames for 10 min: 10 s × 12, 30 s × 2,60 s × 1,6 min × 1). All acquired images were resliced in short-axis and vertical and horizontal long-axis orientations.

The functional parameters end-systolic volume (ESV), end-diastolic volume (EDV), and ejection fraction (EF) were automatically derived from rest and stress ECG-gated acquisitions. The TID was calculated automatically by CardIQ Physio (GE Healthcare) by determining the ratio of the stress endocardial volume and the rest endocardial volume in static non-gated images.

The perfusion defect scores, including summed stress score (SSS), summed rest score (SRS) and summed difference score (SDS), were automatically analyzed using the QPS software (Cedars-Sinai Medical Center, Los Angeles, CA) based on a 20-segment model following the guideline of American Heart Association for each rest and stress study[12]. The summed difference score (SDS) was computed by subtracting the SSS from the SRS. A PET scan was considered normal myocardial perfusion if SSS  ≤ 3 and abnormal myocardial perfusion if SSS > 3[13].

The dynamic images were analyzed by the HeartSee software package (version 3, USA, FDA 510(k) approval K171303) to generate the absolute myocardial perfusion[14]. The software used a simplified retention approach that automatically calculated myocardial blood flow (MBF, mL/min/g) at rest and during vasodilator stress. Myocardial flow reserve (MFR) was calculated as the ratio of stress to rest MBF and considered reduced when MFR < 2 [15].

### Outcome

Patients were followed from the initiation of ^13^N-Ammonia PET until the last day of follow-up (November 22, 2022), or until a prespecified outcome occurred. The observed endpoint was a composite of MACE, which included re-hospitalization for heart failure or unstable angina, late revascularization including PCI or CABG (occurred more than 90 days after PET/CT scan), non-fatal myocardial infarction (MI), and cardiac death. The data of all patients were obtained from electronic health records or followed by telephone contacts with patients or their families. The term "survival" in the study refers to the duration of time between the ^13^N-Ammonia PET examination and either the initial occurrence of MACE or the predetermined end date of the follow-up period.

### Statistical analysis

All categorical variables were tested by ${x}^{2}$ test and the results were reported as frequencies (%). All continuous variables were tested by *t*-test or Mann–Whitney *U*-test depending on the test results of normal distribution and homogeneity of variances and reported as mean (± SD) or median (interquartile range).

The optimal cut-off value for the TID ratio was calculated using receiver operating characteristic (ROC) curve analysis based on the incidence of MACE in patients. Patients were categorized into two groups according to normal and abnormal TID. To investigate the correlation between TID and other continuous variables, the Pearson coefficient with Fisher r-to-z transformation for significance was applied. The event-free survival curve stratified by the TID cut-off value was estimated with the Kaplan–Meier survival methods and compared by log-rank test. Furthermore, multivariable Cox regression analysis was performed to identify predictors of MACE for all patients and subgroups of patients with SSS ≤ 3, and SSS > 3[16, 17]. Variables were transformed into dichotomous variables if needed.

A *P* value < 0.05 was considered to indicate statistical significance. All analyses were performed using GraphPad Prism 9.5.0 (GraphPad Software Inc., San Diego, CA, USA) and R studio (version 4.1.1, http://www.R-project.org; The R Foundation for Statistical Computing, Vienna, Austria).

## Results

### Patient characteristics and outcome

**Fig. 1:**
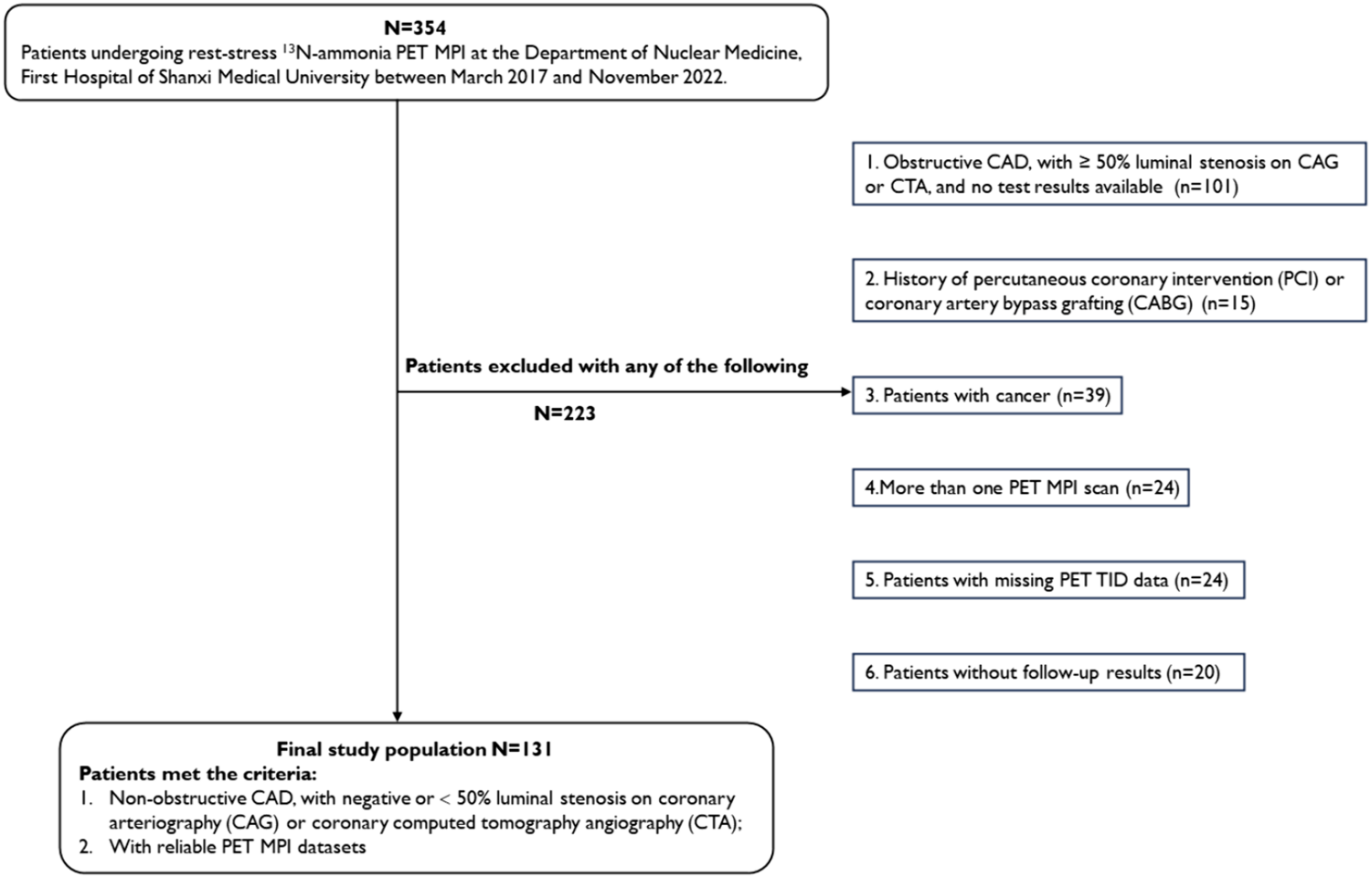
Flow diagram of the study cohort. *PET* positron emission tomography, *MPI* myocardial perfusion imaging, *CAD* coronary artery disease, *CAG* coronary arteriography, *CCTA* coronary computed tomography angiography, *PCI* percutaneous coronary intervention, *CABG* coronary artery bypass grafting, *TID* transient ischaemic dilatation, *MACE* major adverse cardiac events

Out of the 354 patients identified, we excluded 223 patients for reasons outlined in Fig. 1. The final study cohort comprised 131 patients, with a mean age of 53 ± 9 years and 74 (56.5%) males. Over a median follow-up period of 42.08 ± 17.67 months, 22 (16.7%) patients occurred MACE. Among them, 18 (13.7%) had readmission, 2 (1.5%) patients had late revascularization, 1 (0.7%) patient had non-fatal MI, and 1 (0.7%) patient had cardiac death.

The baseline characteristics of patients with and without MACE are listed in Table 1. There were no significant differences in terms of age, sex, body mass index, and cardiovascular risk factors between patients who experienced with and without MACE. Upon comparing the medication’s usage of both groups, the patients with MACE observed a higher usage of ACEI/ARB, Anti-platelet, and Nitrates (all with a significance level of *P* < 0.05), while no significant differences were found for CCB, β-blocker, and statins.

**Table 1:** Baseline characteristics of the study cohort

Characteristic	Total	Without MACE	With MACE	*P* value
	(*n* = 131)	(*n* = 109 [83.2%])	(*n* = 22 [16.8%])	
Age (years)	53 ± 9	53 ± 9	52 ± 8	0.779
BMI (kg/m^2^)	25.71 (23.73–27.68)	25.64 (25.49–27.68)	25.9 (24.24–28.90)	0.359
Male	74 (56.5)	61 (56.0)	13 (59.1)	0.787
Smoking	50 (38.2)	39 (35.8)	11 (50.0)	0.21
Diabetes mellitus	33 (25.2)	25 (22.9)	8 (36.4)	0.186
Hypertension	61 (46.6)	48 (44.0)	13 (59.1)	0.197
Hyperlipidemia	79 (60.3)	65 (59.6)	14 (63.6)	0.726
Family History of CAD	30 (22.9)	22 (20.2)	8 (36.4)	0.099
Medications				
ACEI/ARB	37 (28.2)	27 (24.8)	10 (45.5)	0.049*
CCB	38 (29.0)	30 (27.5)	8 (36.4)	0.405
*β*-blocker	32 (24.4)	24 (22.0)	8 (36.4)	0.153
Anti-platelet	47 (35.9)	35 (32.1)	12 (54.5)	0.045*
Statins	65 (49.6)	52 (47.7)	13 (59.1)	0.33
Nitrates	7 (5.3)	3 (2.8)	4 (18.2)	0.012*
Diuretics	6 (4.6)	4 (3.7)	2 (9.1)	0.267

*BMI* body mass index, *ACEI* angiotensin-converting enzyme inhibitors, *ARB* angiotensin receptor blockers, *CCB* calcium channel blockers

*Statistically significant difference between groups

### Correlation between TID and LV volumes, LV function, perfusion defect scores, global MBF, and MFR

**Fig. 2:**
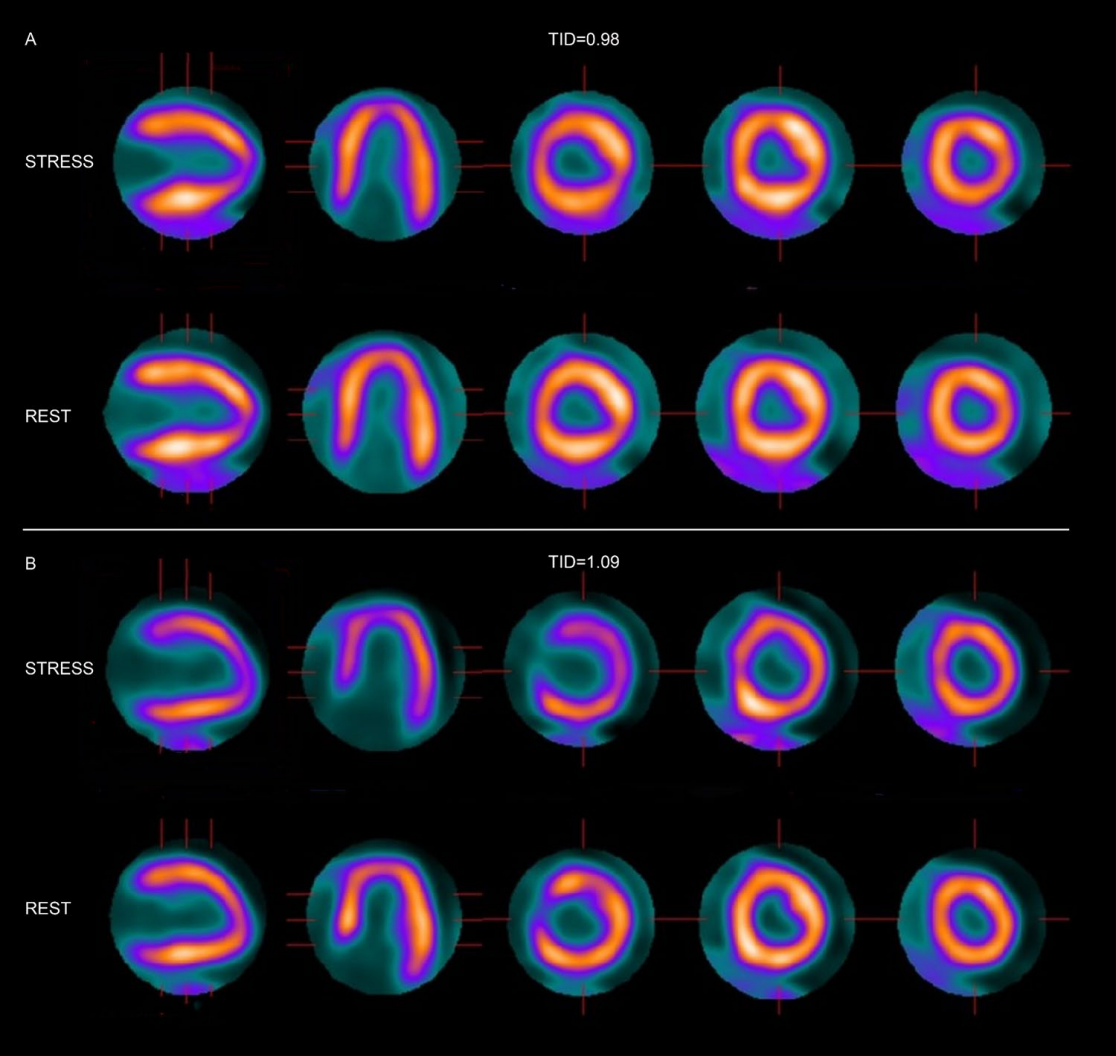
Two typical cases of TID-normal and abnormal. **a** An adult patient in their 40s, whose ESV is 40ml and EDV is 104ml during stress, ESV is 44ml and EDV is 97ml during rest, with normal TID (0.98), who did not occur MACE during the follow-up period. **b** An adult patient in their 70s, whose ESV is 54ml and EDV is 115ml during stress, ESV is 48ml and EDV is 102ml during rest, with abnormal TID (1.09), who occurred MACE during the follow-up period

Table 2 presents the results regarding LV volumes, LV function, perfusion defect scores, global MBF, and MFR for the subgroups with normal and abnormal TID. ESV and EDV were extracted from the LV volumes which were obtained from gated PET images, and showed no significant difference between the TID subgroups stress. However, ESV and EDV at rest were significantly lower in the TID-abnormal group compared to the TID-normal group (ESV rest, 42 (35–55) vs. 36 (30–47), *P* = 0.003; EDV rest, 95 (84–112) vs. 82 (71–99), *P* = 0.004). There was a significant difference in the change of ESV (ΔESV [stress-rest]) and EDV (ΔEDV [stress–rest]) between stress and rest. ΔESV (stress–rest) and ΔEDV (stress–rest) were significantly higher in the TID-abnormal group compared to the TID-normal group (ΔESV [stress–rest], − 4 (− 6 to − 1) vs. 3 (0 to 6), *P* < 0.001; ΔEDV [stress–rest], 9 ± 7 vs. 16 ± 7, *P* < 0.001). Though the EF showed no significant difference between the TID subgroups during stress or at rest, the EF reserve (ΔEF [stress–rest]) was significantly smaller in the TID-abnormal group (ΔEF [stress–rest], 8 (5 to 10) vs. 3 (0 to 7), *P* < 0.001). Perfusion defect scores revealed no significant differences for SSS, SRS, and SDS. Similarly, global MBF at rest and during stress, as well as the MFR did not show significant differences between TID-normal and TID-abnormal patients. The representative cases in the TID-normal and abnormal groups are shown in Fig. 2.

**Table 2:** Distribution of functional parameters in patients with normal TID (< 1.03) as compared to abnormal TID (≥ 1.03)

Variable	Total	Normal TID	Abnormal TID	*P* value
	(*n* = 131)	(*n* = 81 [61.8%])	(*n* = 50 [38.2%])	
EDV rest (ml)	90 (78–109)	95 (84–112)	82 (71–99)	0.004*
EDV stress (ml)	103 (89–119)	104 (90–124)	99 (86–116)	0.408
△EDV (stress-rest)	12 ± 7	9 ± 7	16 ± 7	< 0.001*
ESV rest (ml)	41 (33–51)	42 (35–55)	36 (30–47)	0.003*
ESV stress (ml)	39 (33–49)	39 (33–49)	39 (32–53)	0.953
△ESV (stress-rest)	− 2 (− 5–3)	− 4 (− 6–1)	3 (0–6)	< 0.001*
EF rest (%)	55 (52–58)	54 (51–58)	55 (52–60)	0.103
EF stress (%)	61 (57–65)	62 (59–65)	60 (55–64)	0.095
△EF (stress-rest)	6 (3–9)	8 (5–10)	3 (0–7)	< 0.001*
SSS	4 (2–6)	4 (2–6)	5 (1–7)	0.679
SRS	1 (0–2)	1 (0–2)	0 (0–2)	0.389
SDS	3 (1–4)	1 (3–4)	1 (3–5)	0.769
MBF rest	0.93 (0.79–1.12)	1.12(0.93–1.35)	1.13 (0.93–1.229)	0.802
MBF stress	2.99 ± 0.92	3.05 ± 1.04	2.97 ± 0.86	0.66
MFR	3.14 ± 0.81	3.17 ± 0.84	3.11 ± 0.92	0.67

*ESV* end-systolic volume, *EDV* end-diastolic volume, *EF* ejection fraction, *SSS* summed stress score, *SRS* summed rest score, *SDS* summed difference score, *MBF* myocardial flow reserve, *MFR* myocardial flow reserve

*Statistically significant difference between groups

**Fig. 3:**
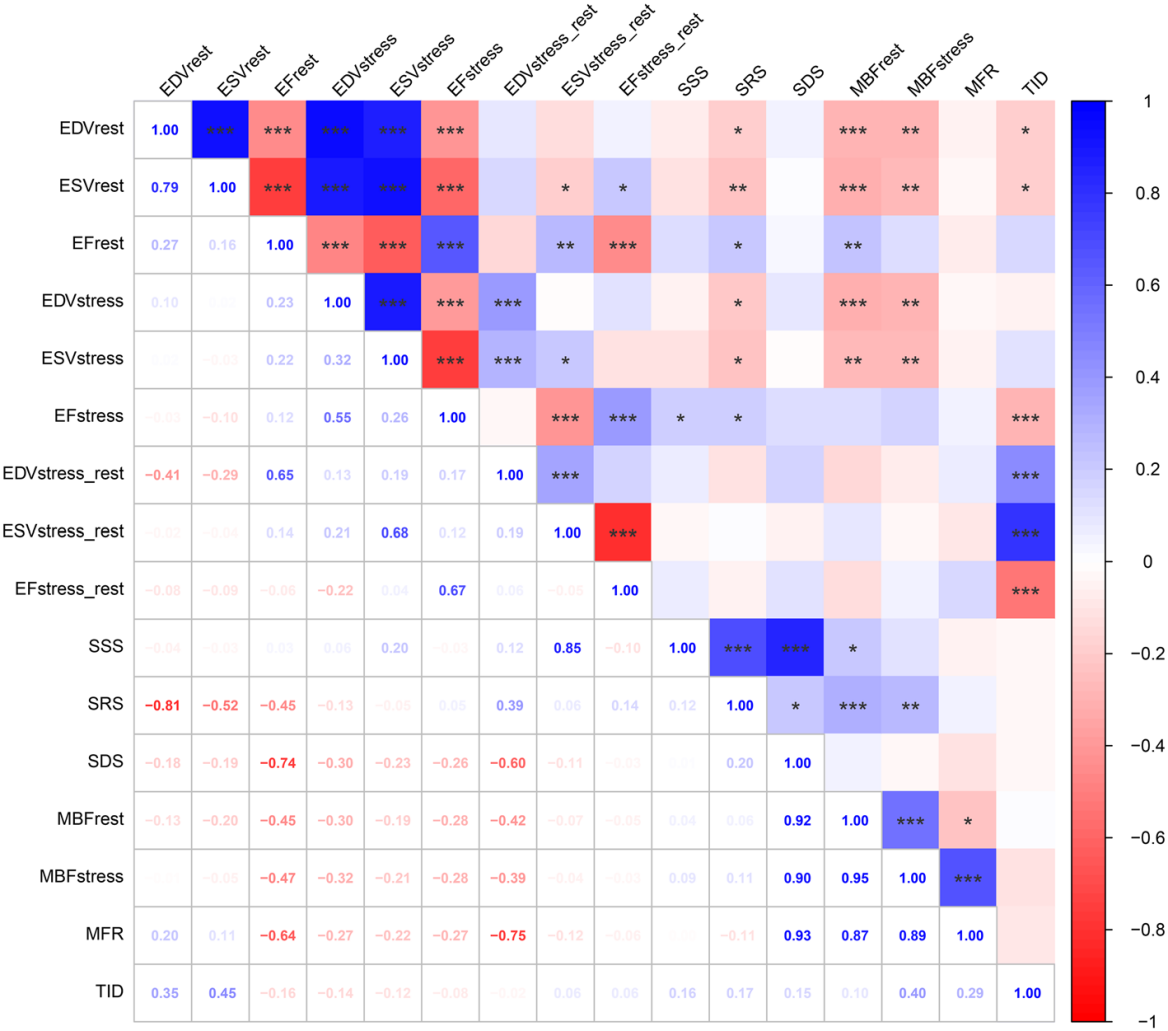
The correlation between TID and other parameters. * *P* < 0.05, ** *P* < 0.01, *** *P* < 0.001. *ESV* end-systolic volume, *EDV* end-diastolic volume, *EF* ejection fraction, *SSS* summed stress score, *SRS* summed rest score, *SDS* summed difference score, *MBF* myocardial flow reserve, *MFR* myocardial flow reserve

ESV and EDV volumes during stress demonstrated no significant correlation with TID (Fig. 3). However, ESV and EDV volumes at rest, ΔESV (stress–rest), and ΔEDV (stress – rest) were significantly correlated with TID (all *P* < 0.05). Although there was no significant correlation between the TID and EF during stress, EF at rest and EF reserve were significantly correlated with TID (both *P* < 0.001, Fig. 3). Furthermore, no significant correlation was detected between TID and perfusion defect scores, MBF, or MFR.

### Outcome analysis

**Fig. 4:**
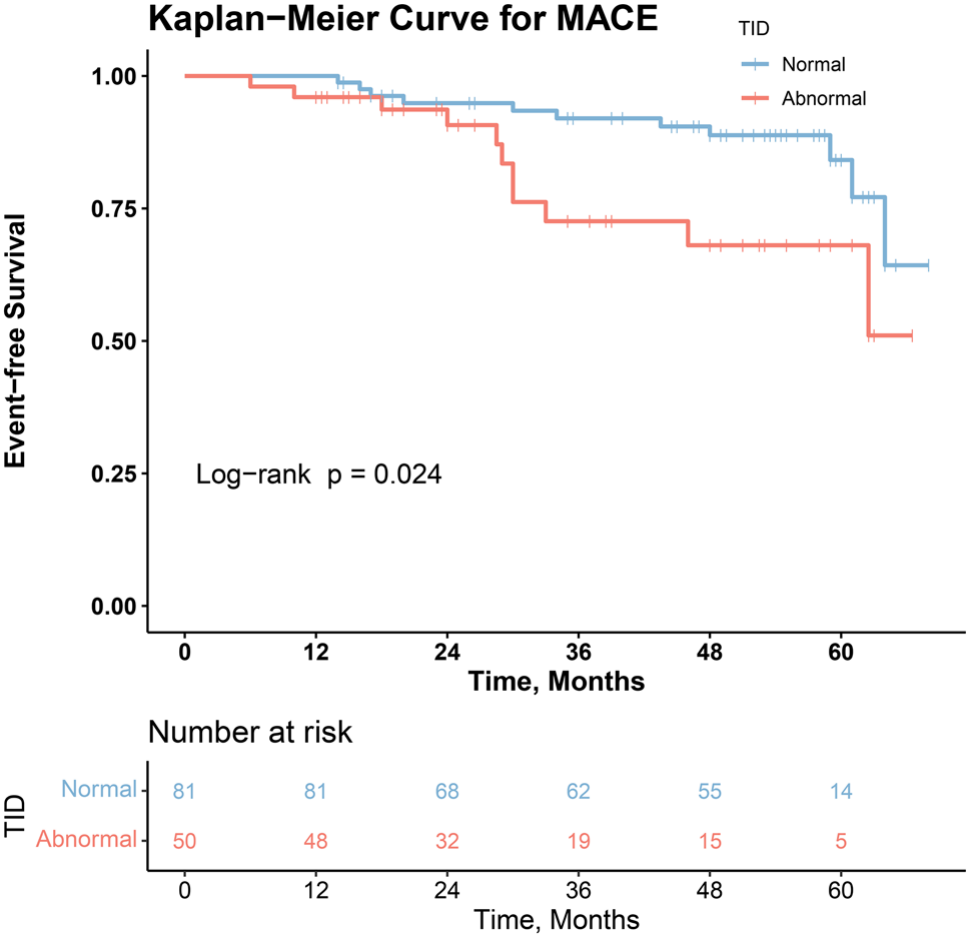
Kaplan–Meier Event-free Survival Curve in the overall study population

**Fig. 5:**
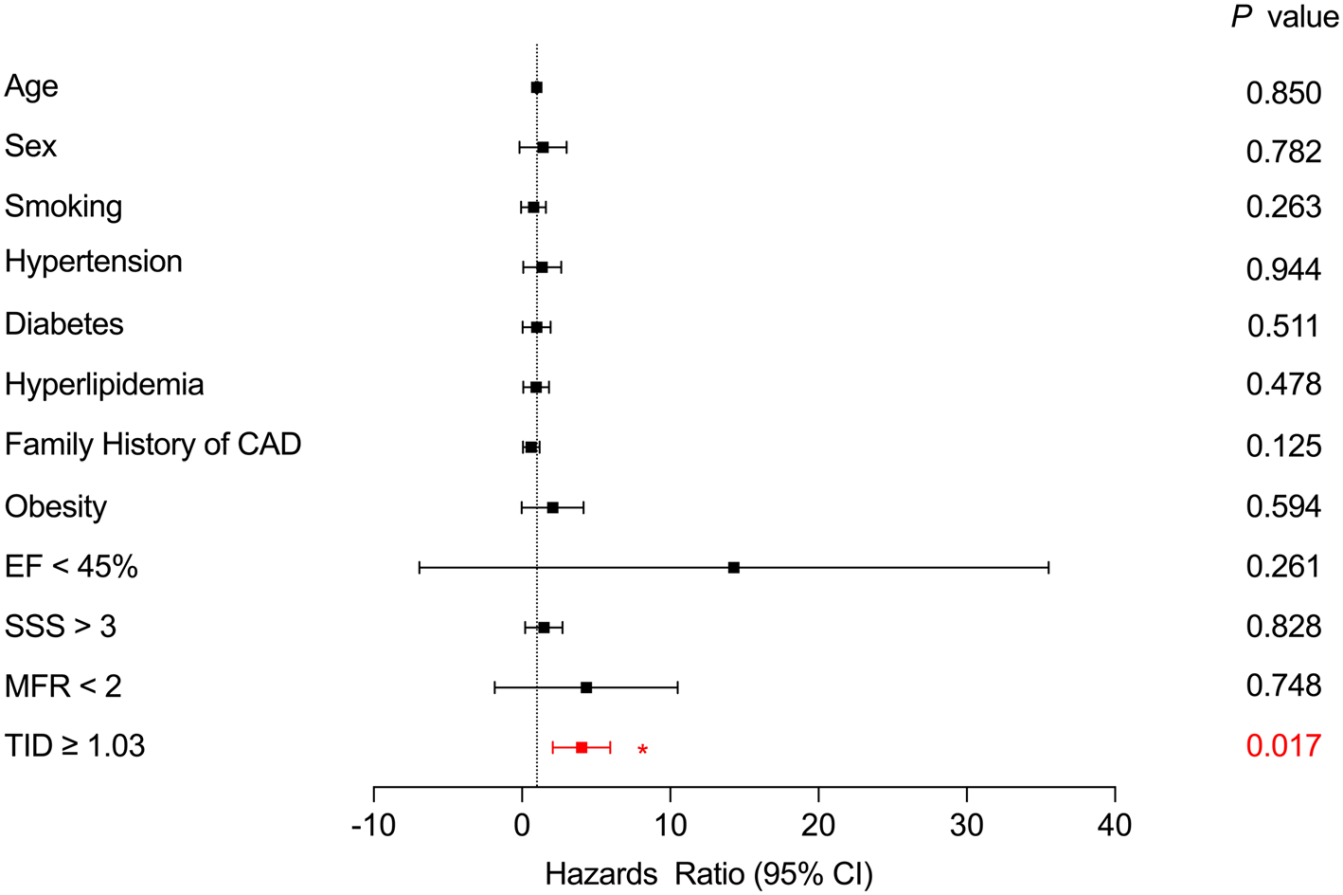
Predictors of MACE in multivariate Cox regression analysis in the overall study population. *EF* ejection fraction, *SSS* summed stress score, *MFR* myocardial flow reserve, *CI* confidence interval

**Fig. 6:**
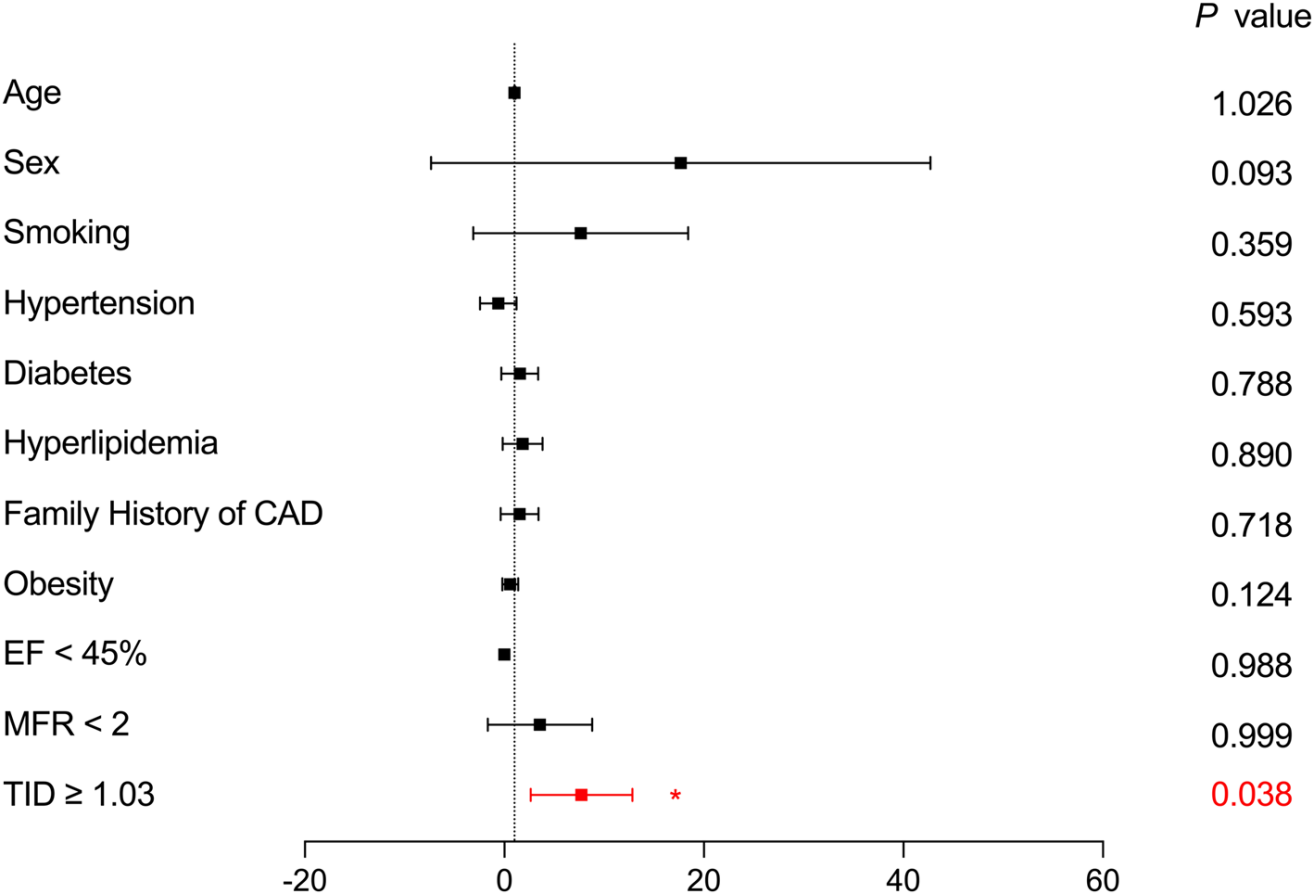
Predictors of MACE in the subgroup of abnormal perfusion. *EF* ejection fraction, *MFR* myocardial flow reserve

Kaplan–Meier survival analysis showed that the MACE-free survival probability was significantly lower in TID-abnormal patients (log-rank = 5.121, *P* = 0.024, Fig. 4). Subsequently, multivariate Cox regression analysis was performed. The factors taken into account were age, sex, conventional cardiovascular risk factors, functional LV impairment (EF < 45%), elevated ischemic burden (SSS > 3), reduced MFR (MFR < 2), and a TID higher than 1.03. The only significantly independent predictor for an event in this analysis was a TID higher than 1.03 (*P* = 0.017, Fig. 5). Furthermore, Cox regression analysis for the subgroups of patients with and without regional perfusion abnormalities (SSS > 3 and ≤ 3) was conducted, which revealed that abnormal TID was an independent predictor in patients with abnormal perfusion patterns (Fig. 6), but not in the subgroup of normal perfusion patterns (Supplementary data, Figure S1).

## Discussion

This study is the first to investigate the prognostic significance of TID in patients with non-obstructive CAD by employing a 1 day rest–stress ^13^N-ammonia PET MPI protocol. The optimal cut-off value of TID is 1.03 by ROC analysis. Patients with an elevated TID show a lower EF reserve. Also, in patients with an abnormal TID, ESV and EDV show a greater rise from rest to stress. The outcome analysis suggests that TID-abnormal subjects have a lower overall survival probability. Furthermore, the multivariate analysis reveals that the abnormal TID is the only independent predictor for MACE in non-obstructive CAD. An abnormal TID is not associated with adverse prognosis in patients with normal perfusion, but it is an independent predictor for MACE in patients with abnormal perfusion patterns, as shown in the subgroup analysis.

TID, the visualization of an increased post-stress LV cavity size compared to rest, is a marker of ischemia in myocardial perfusion imaging. This phenomenon has been associated with both global subendocardial ischemia and post-stress stunning. Notably, MACE events are primarily driven by re-hospitalization for heart failure or unstable angina in our study. Both of them are associated with myocardial ischemia due to impaired LV myocardial function, which may also be relevant to the development of TID.

TID can typically be quantified by calculating the ratio of the post-stress to rest non-gated LV cavity volumes. In this study, the optimal TID cut-off value identified was 1.03 in ^13^N-ammonia PET imaging, which is slightly lower than previously established normal values of TID in SPECT imaging, which are 1.12 to 1.52 [2, 18]. The reasons could be variances in imaging protocol, study population, and threshold calculation method. In contrast to the conventional SPECT acquisition of perfusion images in 15–60 min after stress, stress images using ^13^N-ammonia are acquired at the peak of pharmacologic stress and at the time of tracer injection.[19] Thus, patients have an elevated heart rate during acquisition and a reduced LV cavity size. Of note, the TID cut-off value in our study was utilized to predict MACE in non-obstructive CAD, contrasting with previous studies that focused on diagnosing severe obstructive CAD,[20, 21] which might also explain the fact that our TID threshold is slightly lower. Additionally, the TID threshold was determined based on the ROC curve analysis in this study, while the mean TID + 2SD or mean TID + 1SD approach was employed in most previous studies[19, 22]. In a recent study, Sim et al. derived TID cut-off values by studying patients with normal MPI and coronary artery calcium scores of zero using a cadmium-zinc-telluride (CZT) camera [23]. Further studies involving larger cohorts of patients who are receiving CZT-SPECT MPI are needed to validate these TID cut-offs.

So far, the role of TID derived from PET MPI has been only marginally investigated. Shi et al. previously suggested an upper normal limit of 1.15 in ^82^Rb PET and demonstrated elevated TID index was a specific, marker of single and multiple vessel CAD [24]. However, they did not provide information regarding the analyses of clinical variables, LV function, MFR, or outcome. In line with our study, Rischpler et al. demonstrated that patients with an elevated TID (≥ 1.13) had more severe LV dysfunction and were at higher risk for MACE [19]. In contrast to our findings, they showed that lower MFR and increased perfusion defect scores can be observed in patients with an elevated TID. This suggests that the presence of TID may be associated with CMD, reflect subendocardial ischemia, and predict poor prognosis in patients with CAD. In contrast to their study, which included patients with known CAD and intermediate or high likelihood of CAD, our study was conducted exclusively in patients with non-obstructive CAD in whom obstructive stenotic lesions were excluded based on the CCTA or CAG examination results. Therefore, the differences in the study populations and the limited sample size contributed to the fact that our study did not show similar results. Nevertheless, based on the understanding of the pathophysiologic mechanisms of TID and the findings of previous studies, it seems reasonable to hypothesize that there may be a correlation between abnormally elevated TID and CMD in patients with non-obstructive CAD. Similarly, other indicators such as papillary muscle ischemia (PMI) on ^13^N-ammonia PET images reflect microcirculatory impairment. Using ^13^N-ammonia PET, Nakao et al. demonstrated that PMI was associated with a reduced global MFR and higher rates of MACE, and it was also an important maker of risk stratification in patients with known or suspected CAD [25]. More importantly, PMI may be a predictor in assessing microcirculatory impairment in ischemia with no obstructive coronary arteries (INOCA), which may reflect subendocardial microvascular ischemia. Sakai et al. showed PMI was frequently detected in patients with INOCA, and the global MFR was significantly lower in patients with INOCA than in those with or without CAD. The presence of PMI and decreased MFR were characteristic ^13^N-ammonia PET findings of INOCA, which hold significant prognosis value in INOCA [26]. Future studies may yield additional insights into the CMD and TID findings in patients with non-obstructive CAD.

Previous studies have revealed that TID is a key predictor for adverse outcomes in SPECT as well as in planar scintigraphy [27–29]. To the best of our knowledge, there is no data available so far on the prognostic implications of TID by ^13^N-ammonia PET in patients with non-obstructive CAD. Our data suggest that an abnormal TID is an independent predictor for MACE in patients with non-obstructive CAD, other conventional cardiovascular factors, EF < 45%, SSS > 3, and MFR < 2 are not, though limited by a limited number of incidents. The MFR cut-off values between 2.0 and 2.5 are not certain in different studies. We take reduced MFR (MFR < 2) as a factor in our regression Cox analysis, as prior studies have demonstrated that an MFR cut-off of 2 is useful for risk prognostication and discrimination of incident cardiovascular outcomes [30, 31].

The relationship of TID and MACE varied depending on normal or abnormal perfusion patterns. Our subgroup analysis indicated that an abnormal TID was not an independent predictor in patients with normal perfusion. The reason for aberrant TID in patients with normal perfusion is yet to be explored. Align with most contemporary studies on TID [22, 32], our finding that an abnormal TID did not provide any additional prognostic value in patients with normal perfusion, was different from those of studies that found higher cardiac event rates in patients with normal(SSS = 0) or near normal (SSS ≤ 3) perfusion [28].The decreasing incidence of severe CAD or cardiac events in patients with TID and normal perfusion in contemporary compared to older studies is believed to be a potential reason[33]. Of note, in our subgroup of abnormal perfusion patterns, an abnormal TID is an independent predictor for MACE, which is identical to the results derived from regadenoson MPI [34].

The optimal medical treatment strategy for patients with non-obstructive CAD remains uncertain. The primary cardiovascular risk prevention guidelines indicate that the intensity of treatment should be based on the clinical risk profile. Further studies with larger sample sizes are required to investigate the specific mechanism of occurrence of abnormally elevated TID in patients with non-obstructive CAD, as well as to determine individualized anti-ischemic therapy based on their individualized clinical risk profiles.

### Limitations

The study has several limitations that should be acknowledged. First, this was a single-center study in non-obstructive CAD, therefore the findings may not be generalizable to other cardiovascular diseases. Secondly, the standard protocol for the TID measurement is still missing, and it was obtained in various settings of SPECT and PET MPI. Thus, the suggested cut-off value of TID in this publication might only be suitable in comparable settings using comparable data acquisition and analysis settings. At last, our data are preliminary because of the relatively small sample size and a low number of events. So more future studies with larger sample sizes are needed to confirm whether 1.03 as the cut-off value is a significant predictive value.

## Conclusion

TID derived from ^13^N-ammonia PET MPI is a critical prognostic factor in patients with non-obstructive CAD, abnormal TID is associated with adverse MACE. In the subgroup with stress perfusion abnormality with SSS > 3, abnormal TID was identified as an independent risk factor for poor prognosis. PET MPI TID is a potential imaging marker for clinical decision-making.

## Supplementary Information

Below is the link to the electronic supplementary material.

Supplementary file1 (DOCX 90 KB)

## Data Availability

Data are available upon reasonable request.

## Abbreviations

CAD: Coronary artery disease
CMD: Coronary microcirculation disorder
CAG: Coronary arteriography
CCTA: Coronary computed tomography angiography
CZT: Cadmium-zinc-telluride
EDV: End-systolic volume
ESV: End-diastolic volume
EF: Ejection fraction
GABG: Coronary artery bypass grafting
MPI: Myocardial perfusion imaging
MBF: Myocardial blood flow
MFR: Myocardial flow reserve
PET: Positron emission tomography
PCI: Percutaneous coronary intervention
SSS: Summed stress score
SRS: Summed rest score
SDS: Summed difference score
TID: Transient ischaemic dilatation
