# Supplementary material for: Prognostic value of transient ischemic dilatation by 13N-ammonia PET MPI for short-term outcomes in patients with non-obstructive CAD
Source: Ann Nucl Med. 2024 Sep 9;39(1):47–57. doi: 10.1007/s12149-024-01976-8 (PMC11706881; doi:10.1007/s12149-024-01976-8)
Supplement: Supplementary file 1 — Supplementary file1 (DOCX 90 KB) [file 12149_2024_1976_MOESM1_ESM.docx]

**Prognostic value of transient ischemic dilatation by ^13^N-ammonia PET MPI for short-term outcomes in patients with non-obstructive CAD**

**Annals of Nuclear Medicine**

*****Yanni jia^1,3^, *****Yingqi Hu^1,4^, Lihong Yang^1,3^, Xin Diao^1,4^, Yuanyuan Li^1,4^, Yanhui Wang^1,4^, Ruonan Wang^1,4^, Jianbo Cao^1,2^, ^#^Sijin Li^1,2^

1. Department of Nuclear Medicine, First Hospital of Shanxi Medical University, Taiyuan, Shanxi, China
2. Collaborative Innovation Center for Molecular Imaging of Precision Medicine, Shanxi Medical University, Taiyuan, Shanxi, China
3. School of Forensic Medicine, Shanxi Medical University, Taiyuan, Shanxi, China
4. Shanxi Key Laboratory of Molecular Imaging, Shanxi Medical University, Taiyuan, Shanxi, China

**^#^** **Corresponding author**

Sijin Li, MD, PhD

Address: Department of Nuclear Medicine, First Hospital of Shanxi Medical University; Collaborative Innovation Center for Molecular Imaging of Precision Medicine, Taiyuan, Shanxi, China

Tel: +8613934519222 Email: lisjnm123@163.com

***First author**

Yanni Jia and Yingqi Hu contributed equally to this work.

Tel: +8618391488382 Email: [jiayanni1012@163.com](mailto:jiayanni1012@163.com)

[Tel: +8619834518792](mailto:Tel:%20+8619834518792) Email: [yingqihu@163.com](mailto:yingqihu@163.com)


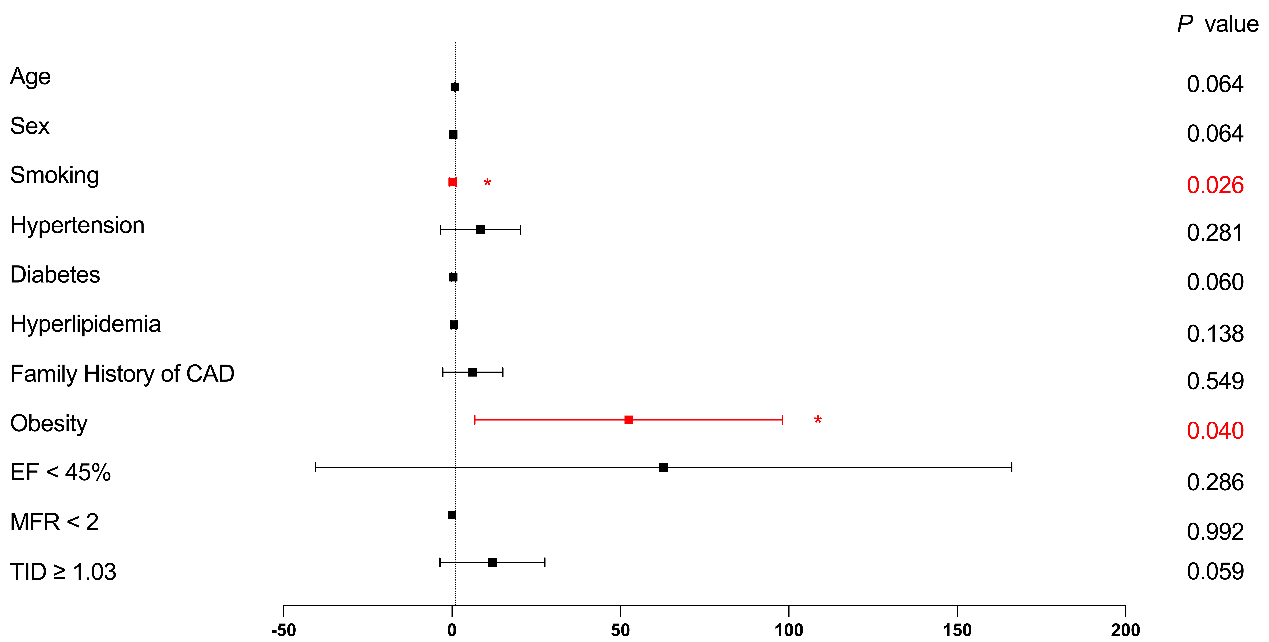
***Figure S1*.** Predictors of MACE in the subgroup of normal perfusion.

EF, ejection fraction; MFR, myocardial flow reserve
